# Responding to a call to action for health equity curriculum development in pediatric graduate medical education: Design, implementation and early results of Leaders in Health Equity (LHE)

**DOI:** 10.3389/fped.2022.951353

**Published:** 2022-10-25

**Authors:** Raquel G. Hernandez, Darcy A. Thompson, John D. Cowden

**Affiliations:** ^1^ Johns Hopkins All Children's Hospital Institute for Clinical and Translational Research, St. Petersburg FL, United States; ^2^ Department of Pediatrics, Johns Hopkins University School of Medicine, Baltimore MD, United States; ^3^ Department of Pediatrics, University of Colorado School of Medicine, Anschutz Medical Campus, Aurora, CO, United States; ^4^ Division of General Academic Pediatrics, Children's Mercy Kansas City, Kansas City, MO, United States; ^5^ Department of Pediatrics, University of Missouri-Kansas City School of Medicine, Kansas City, MO, United States

**Keywords:** health equity, health disparities, graduate medical education, curriculum, leadership, quality improvement

## Abstract

**Introduction:**

Recent calls to action have urged graduate medical education leaders to develop health equity-focused curricula (HEFC) to redouble efforts to promote pediatric HE and address racism. Despite this call, examples of HEFC for pediatric residents are lacking. Such curricula could catalyze educational innovations to address training gaps.

**Objective:**

To describe the design, content, and delivery of “Leaders in Health Equity (LHE),” an innovative HEFC delivered to categorical pediatric residents using multi-modal, service-free retreats.

**Methods:**

This single institution, longitudinal curriculum study occurred between 2014 and 2020 and reports multi-level outcomes including: (1) impact on trainee's health equity related knowledge, skills and satisfaction, (2) residency impact and (3) institutional impact. Educational approaches used related to design, content and delivery are summarized and detailed.

**Results:**

Trainees (*n* = 72) demonstrated significant improvements in pre-post knowledge and skills related to HE content. Residents also reported increased desire for advanced HE content over the course of the 6-year study period. Residency impact on operations and resources were sustainable with the opportunity for integration of LHE content in other curricular and training areas noted. Institutional impact included catalyzing organizational HE initiatives and observing an increase in resident-led quality improvement (QI) projects focused on LHE content.

**Conclusions:**

On-going adaptation and growth of LHE content to educate increasingly prepared pediatric trainees is a critical next step and a best practice for educators in this evolving field. Developing HEFC within pediatric training programs using a longitudinal, leadership-centered approach may be an effective educational strategy in addressing pediatric health disparities.

## Introduction

Training future generations of pediatricians to deliver culturally appropriate care and promote health equity is a graduate medical education (GME) priority redoubled in the context of COVID-19-related racial/ethnic disparities and the increased recognition of racism as a public health crisis (1). Recently, funding agencies including the Robert Wood Johnson Foundation have focused on defining key terms by which to catalyze more efforts addressing health disparities, introducing a definition by which we can specify that *pediatric health equity* reflects a collective goal that “every child has a fair and just opportunity to be as healthy as possible…that requires removing obstacles to health such as poverty, discrimination, and their consequences, including powerlessness and lack of access to good jobs with fair pay, quality education and housing, safe environments, and health care” (2). In a novel call to action, Siegel et al. urged GME educators “to develop curricula that are accountable to community needs and that more comprehensively address health inequities” (3). This training expectation is further highlighted by the Accreditation Council for Graduate Medical Education's (ACGME) Clinical Learning Environment Review (CLER) program, which assesses trainee connectivity to their institution's health equity efforts. Moreover, the American Board of Pediatrics (ABP) has similarly emboldened all pediatricians to engage in health equity efforts by launching new entrustable professional activities (EPAs) focused on creating competency among pediatricians towards addressing racism, discrimination, and other contributors to inequities (4). These newly launched training and certification standards highlight the timely need for health equity-focused curricula (HEFC) as a strategy to promote pediatric health equity. Beyond meeting accreditation and curricular requirements, however, integrating HEFC into pediatric graduate medical education broadens pediatric trainees' ability to recognize and address the social determinants of health and promotes a cultural shift to make health equity efforts the responsibility of all pediatricians.

*Health equity-focused curricula* can be broadly defined as training experiences including courses, rotations and other education providing trainees with the knowledge and skills to identify and ameliorate health disparities in their own patient populations and in the systems in which they work. Early efforts related to HEFC in medical education have demonstrated the value of using retreat-based approaches for engaging emergency medicine trainees in health-equity discussions (5) where residents described being more meaningfully engaged in HE discussions by being briefly relieved of service duties as part of the curriculum design. There is also early evidence of the impact of HEFC on medical student confidence levels and knowledge in working with underserved populations (6). To our knowledge, no prior HEFC have focused on multi-level outcomes among pediatric residents. Innovations related to health equity integration within pediatric GME programs are thus urgently needed to address heightened training expectations and promote systemic health equity strategies to address pediatric racial and ethnic disparities.

Six years ago, we identified a rare opportunity to design and deliver a health equity-focused curriculum while developing a new ACGME-accredited pediatric residency program (Johns Hopkins All Children's Hospital). A central component of the pediatric residency program design was a longitudinal leadership-focused curriculum [Leadership Executive Academic Development (LEAD), described elsewhere] (7) embedded into annual 1–2-week service-free retreats. Viewing this training model as an ideal forum to offer health equity-focused training to residents, we nested a novel curriculum titled “Leaders in Health Equity” (LHE) within the LEAD framework.

We reflect here on our experience in creating and delivering LHE and describe early results related to trainees, the residency program, and institutional impact.

## Methods

The primary goal of LHE was to provide immersive, longitudinal training that allowed pediatric residents to meaningfully engage in health equity-focused content during service-free retreats. LHE was delivered annually where PGY-1 residents engaged in a 1.5- to 2-day sessions. Key LHE principles and approaches related to curriculum design, content, and delivery included the following (Tables 1A–C):

Design: (1) longitudinal format, (2) service-free schedule, (3) requirement for all residents, and (4) integration into leadership (Table 1A).
Content: (1) multiple faculty instructors with subject matter expertise, (2) identification of core topics with commitment to evolving content over time, and (3) connectivity of content to quality improvement (QI) frameworks (Table 1B).
Delivery: (1) use of diverse modalities, (2) time for introspection, (3) immersive design, (4) use of cohorting and small group structure, and (5) multi-level assessments (Table 1C).

**Table 1A:** Leaders in Health Equity (LHE) design principles and detailed approaches.

Design principle	Approach
Longitudinal format	LHE was delivered annually during the 1-week LEAD^a^ retreat. PGY-1 residents engaged in a 1.5- to 2-day experience while PGY-2 and 3 residents engaged in a 1-day experience. Some components were delivered to multiyear resident groups to reflect team structures in clinical rotations. Delivering LHE on a recurring, annual basis allowed for reinforcement and expansion of content year over year.
Service-free schedule	LHE days included intensive 4-hour morning and afternoon sessions free from clinical service responsibilities.
Requirement for trainees	All residents were required to participate and were provided mandatory pre-session reading material (e.g., journal articles, policy statements) and written exercises (e.g., case descriptions, self-assessments).
Integration into leadership training	The inclusion of LHE within the LEAD program conceptually connected health equity into the broader framework of leadership skills, reinforcing the concept of health equity as a universal competency for all physicians.

^a^ LEAD, Leadership Executive Academic Development.

**Table 1B:** Leaders in Health Equity (LHE) content principles and detailed approaches.

Content principle	Approach
Multiple faculty instructors	The LHE curriculum was developed by the 3 co-authors: the local residency program director (RH) and two external faculty (JC, DT) from separate academic centers, each with clinical, educational, and research expertise in health equity topics. Combining the experience and perspectives of three collaborators with long-standing relationships allowed us to create a curriculum with breadth and depth on a range of topics. In addition, the residency program director was able to place broad curricular concepts (e.g., community health disparities, organizational culture) in the context of the residents’ local learning environment.
Inclusion of core topics with evolution	Topics common to all years included culture and communication, healthcare disparities, bias and racism, health literacy, and language support for patients/families with limited English proficiency. Our primary goal from the outset was to assure that the way topics were taught evolved in accordance with learners’ needs and feedback. We added topics over time related to community health needs and institutional priorities, including care of immigrant children, integration of health equity into quality improvement, and an enhanced focus on racism.
Connectivity to quality improvement framework	In years 5 and 6, we introduced the topic of health equity integration into quality improvement (QI) frameworks. We sought to demonstrate that health equity content would help the residency program achieve goals related to ACGME's^a^ CLER^b^ aims and reinforce QI tools and frameworks. In our most recent adaptation of the curriculum, we included content that utilized existing institutional challenges (e.g., disparities in patient outcomes, community health needs assessment) to ensure relevancy to daily patient care and to support resident engagement in solutions related to health disparities.

^a^ ACGME, Accreditation Council for Graduate Medical Education.

^b^ CLER, Clinical Learning Environment Review.

**Table 1C:** Leaders in Health Equity (LHE) delivery principles and detailed approaches.

Delivery principle	Approach
Diverse modalities	LHE included traditional didactic presentations, peer discussions (large and small group), interactive exercises, introspective discussions (e.g., clinical challenges related to culture, case-based discussions, and role play. Hospital interpreters took part in language-related role play). Scenarios to promote resident engagement and self-efficacy, as well as fidelity in role play experiences.
Time for introspection	Opportunities for introspection and debriefing were nested within sessions throughout the retreat (Table 1). This created space for faculty and residents to talk about their biases, habits, and personal and professional challenges related to a variety of topics. The goal was to promote un-pressured and meaningful engagement in identifying and addressing vulnerabilities and perspectives related to diversity, health equity, and inclusion where success was measured in resident engagement level. When LHE was delivered virtually in 2020, we observed a drop-off in the degree of meaningful interaction among all participants, highlighting the potential importance of in-person delivery to previous years’ successes.
Immersive design	The immersive service-free design allowed for individual introspection (e.g., What can I learn about myself and my biases?) and group-level reflection (e.g., What have we learned about our collective group's biases, diversity, and identity as a class within the institution?). Sessions were held offsite or in non-clinical buildings, where residents and faculty could commit their full attention to LHE activities.
Cohorting and group size	Sessions were primarily structured around each class of residents (*n* = 12) or a resident group small enough to participate and receive individual attention from facilitating faculty. Smaller groupings (2–4 residents) were used at times to facilitate trust-building and perspective-sharing. On day 2, case-based discussions including two classes of residents (*n* = 24) encouraged interplay based on shared clinical experiences and resident team interactions.
Multi-level session assessments	Residents completed knowledge and skill assessments 1-week pre and 1-week post LHE. Residents additionally completed formative session evaluations at the close of each day including open-ended qualitative comments. LHE faculty-facilitators rotated responsibility of taking notes on session timing, interactivity, and resident comments/questions, which were used annually to assess topic relevance and effectiveness of content delivery. LHE content was regularly modified and evaluated based on these multi-level assessments.

Although broad curricular goals remained the same over 6 years, we adapted content and delivery annually (Table 2). Learning objectives for LHE were initially focused around understanding the clinical needs of limited English proficient populations, the role of stereotypes and unconscious bias in clinical care, as well as the role of language in clinical care (Table 3). These learning objectives evolved over the 6-year study period, with new concepts (e.g., the role of system-based biases, use of quality improvement to address disparities, integration of health equity within day-to-day efforts) added over time. Data on trainee knowledge/skills and session feedback were collected and analyzed annually. This study was deemed exempt by the Johns Hopkins Medicine Institutional Review Board.

**Table 2:** Leaders in Health Equity (LHE) curricular content evolution.

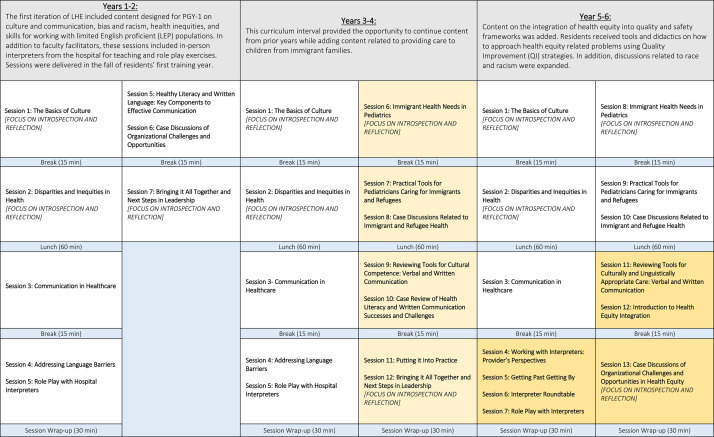

Daily session agendas and topics, years 1-6 (Gold denotes revisions of previous content).

**Table 3:** Leaders in Health Equity (LHE) learning objectives.

• Define key concepts of culture and ethnocentrism • Define Limited English Proficiency (LEP) • Understand the unique needs of the LEP population, including: • form a therapeutic relationship with LEP families • communicate effectively with LEP families • assess family's language preference • assess family's understanding of the care plan • identify whether an interpreter is needed • assess whether LEP family can read handout material
• Describe best practices to address language barriers and communication challenges • Describe disparities and the major factors that influence health • Define health disparities within the local and regional community • Understand the societal and cultural influence on care delivery • Define bias, stereotyping, discrimination and racism • Describe how bias and stereotyping can affect medical decision and care • Understand the concept of Health Equity Integration (HEI) • Understand the role of quality improvement in addressing identified health disparities

### Statistical analyses

Pre/post scores for survey items were summarized with medians and ranges. Data were first evaluated by year and subsequently pooled across years. Given the non-normal distribution of scores, Wilcoxon's signed-rank test was used to evaluate differences. *P *< 0.05 was considered statistically significant. Statistical analyses were conducted using Stata/SE Version 17.1 and accompanying graphs were created using GraphPad Prism Version 8.0.1 for Windows (GraphPad Software, San Diego, California USA, www.graphpad.com).

## Results

**Figure 1:**
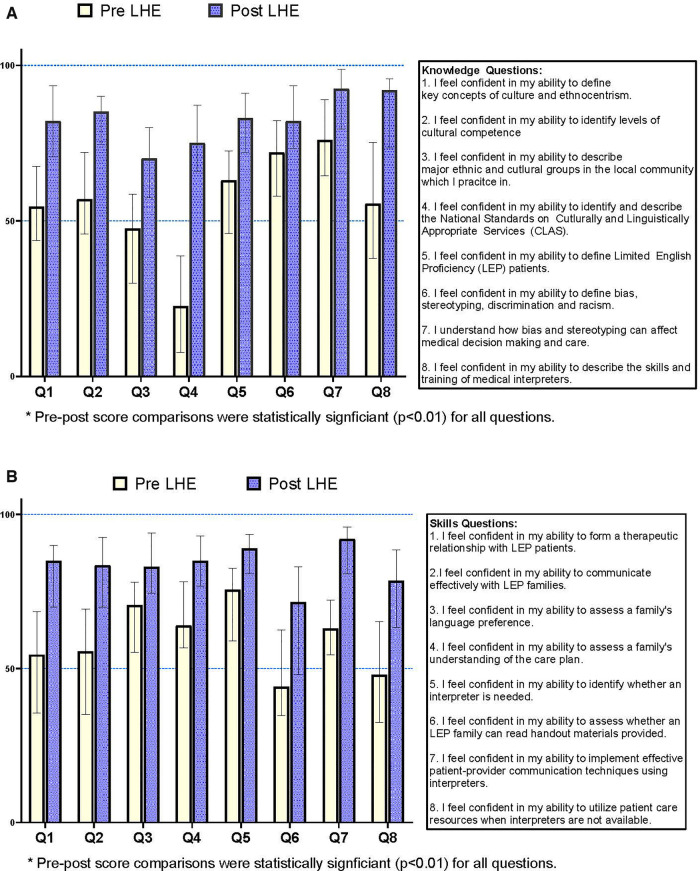
(**A**) Resident self-reported median confidence score (Interquartile Range): LHE knowledge*. (**B**) Resident self-reported median confidence score (Interquartile Range): LHE skills*.

1. Trainee Impact
  • Over 6 years, 120 pediatric residents participated (increasing from 12 residents/year in years 1 and 2 to 36/year after the program had grown to 3 classes). We report data on PGY-1 residents completing LHE training (*n* = 72).
  • Knowledge and Skills: We surveyed residents using an electronically based 16-question survey (2014–2017, pre-*n* = 44 [57% response rate], post *n* = 28 [39% response rate]). Residents were asked to rate their confidence and knowledge related to key questions using a visual ruler scale (scale: 0%-100%). We analyzed pre-post median responses for a series of 8 health equity-focused questions on knowledge and skills respectively (Figures 1A,B). Statistically significant changes were noted across all knowledge and skills questions with the greatest reported change in confidence noted regarding understanding the skills and training of medical interpreters (knowledge) and being able to identify whether an LEP family can understand written handout materials (skills). A new pre-post question format was introduced in 2018, limiting direct comparisons to prior quantitative data.
  • Session feedback: Residents completed free-text survey questions following LHE sessions describing overall satisfaction and areas of potential improvement. In all years, residents gave high ratings to LHE sessions including peer-peer discussions, interactivity and role-play, and a focus on introspection and self-awareness. We noted an evolution in resident preparedness related to health equity, with those in more recent years requesting more advanced content, commenting that multiple health equity topics had been introduced in medical school.
2. Residency Impact
  • Operations and resource impact: As a categorical program of 12 residents per class, we were able to deliver this service-free, annual, 1.5- to 2-day retreat using volunteer faculty to cover inpatient units. Costs for LHE curricula included (1) annual honoraria and travel costs for two external faculty, (2) fees for 3–4 interpreters to join session(s) ranging from $50–60/hour/interpreter, (3) catering (breakfast and lunch for 12 residents; $1000/day), and (4) non-clinical spaces (two local hotel conference rooms [$200/day]; on-campus educational space [no cost] in LHE years (5–6).
  • Curricular impact: As a result of annual LHE workshops, content on disparities, health equity, and culturally/linguistically appropriate care has been integrated into multiple areas of the residency curriculum allowing residents to repeatedly revisit key concepts throughout training. Experiences include annual standardized patient scenarios focused on social determinants of health, research-based health equity sessions, and completion of a community rotation focused on underserved populations. Additionally, residents revisit LHE topics during quarterly conference sessions where residents lead small groups in reviewing health equity-related cases.
3. Institutional Impact
  • Early on, residents outlined concerns regarding their learning environment including inadequate on-site interpreter access, biases observed within patient care, and personal experiences with bias and prejudice. In response, residents and program leadership collectively initiated an institutional effort to submit safety reports related to insufficient in person language support. This reporting prompted a broader language support services assessment (2017–2020) that led to improved in-person language services in the hospital. In addition, the number of resident-led quality improvement projects per year focusing on questions of health equity has grown annually, from 10% to 30% of projects. Finally, 4 trainees achieved language proficiency certification in languages other than English, a resource introduced and promoted through LHE workshops and subsequently offered by the institution to bilingual physicians.

## Discussion

Our innovative longitudinal LHE curriculum, demonstrating multi-level impacts, provides an example of how to meet recent calls to action from GME leaders (3) to develop health equity-focused curricula. As GME programs look to increase and improve training in health equity and related concepts, new models are needed that move beyond didactic lectures, single workshops, and other short-term approaches and pediatric trainee programs may be best primed to lead these educational charges for change. Longitudinal integration of health equity content into existing competency goals (such as leadership development and quality improvement) is needed to avoid such content being perceived as “extra work” or “separate” from fundamental professional development. Leadership tenets offered in the LEAD framework such as self-reflection, creating change, and improving the quality of patient care (8), are principles that naturally extend to health equity calls to action. As Wright et al. proposed in their health leadership competency framework, a physician should “become[e] a change agent that models and facilitates the integration of cultural humility and cultural competency… [into daily practice]”(6).

A key strength to our curricular approach is integrating health equity content with a leadership training framework and quality improvement approaches. These frameworks inherently promote a focus on trainee self-introspection, life-long learning, problem-solving, and commitment to promoting equity that are necessary components to initiating a health equity mindset that may persist beyond completion of the training experience and be sustained throughout training. In our experience, educational efforts addressing cultural competency, disparities, and health equity often are one-off lectures focused on knowledge and attitudes, lacking the practical skill-building over time that is necessary to advance health equity. Other strengths to our approach include (1) a consistent retreat-based framework housing flexible content that can be adapted year-to-year in response to rapidly changing trainee readiness, (2) multiple instructors offering current examples and experience in practical health equity work (rather than only theoretical concepts), and (3) sustained multi-level impacts from a modest investment of resources.

While the application of our model in a single pediatric residency limits the generalizability of our findings, the key elements to our approach, the combination of topics in our curriculum, and the lessons learned from our integrative model could be instructive for others aiming to make health equity a fundamental part of GME, rather than an add-on or bonus topic. Additionally, core learning objectives reflected the application of essential health equity topics to local circumstances, a tactic central to our integrative approach. The resulting curriculum is unique, making it less applicable in another context without modification, but making it specifically relevant to our learners in a way that “off-the-shelf” curricula are not. We believe that educators in each learning context should consider a similar process, where the essential topics listed in our curriculum might be applied to their own specific circumstances and modified year-over-year to reflect rapid changes in trainees and health equity practice. Our quantitative trainee data is limited by sample size and variable response rates, common challenges in residency training evaluation. However, we could identify trends in learner responses that guided the evolution of our model over 6 years. Finally, knowledge and skills were assessed using an unvalidated program-specific survey. Because the primary intent of the surveys was to provide grounding in trainees’ health equity gaps, they have served their purpose to date. The opportunity to enhance the rigor and validity of LHE outcomes is a focus of our team's next steps.

Based on our experiences with LHE, we offer the following recommendations that reflect our own next steps and provide strategies that may be helpful to others embarking on health equity curriculum development:

1. **Routinely engage pediatric trainees in ongoing modification of health equity content development, implementation, and delivery**. The effectiveness of LHE programming has relied on our ability to incorporate resident feedback, new developments (e.g. current racial justice movements), and lessons learned from previous years (e.g. organizational health equity efforts). We meet periodically throughout the year to modify and add to the curriculum for the upcoming fall. Early in the COVID pandemic, we discussed improvements to our virtual learning approach and the incorporation of COVID disparities, anti-racism movements, and rapidly-evolving US immigration policies that impact child health. Input from residents has been critical and has reflected progressively increasing interest, comfort, and preparation in areas related to health equity.
2. **Seek practical tools and deliberate approaches to move diversity, equity, and inclusion efforts from the abstract to the clinically applicable including quality improvement.** The inherent connectivity of health equity and QI was recently highlighted by Ayosla et al., who described how QI aims focused on addressing disparities pose a “win-win” for patients and educators (9). One of the two external LHE faculty (JC) first introduced a checklist-oriented framework for integrating diversity, equity, and inclusion (DEI) perspectives into QI into the 2018 program. This framework was well received and will be further implemented in future sessions to promote the universal inclusion of DEI in resident QI efforts.
3. **Adapt or create methods for directly observing skills taught in health equity curricula.** Recognizing that gains in trainee knowledge are not equivalent to behavior change that impacts patient care, we plan to use new evaluation methods (incorporating competency-based assessments) to understand how trainee perceptions, biases, understanding of health disparities, and clinical skills may shift as a consequence of this training. Promising progress has been made in the field using DEI-related simulations and behavior-based evaluation such as objective structured clinical encounters (OSCE's) involving culturally or linguistically challenging scenarios (10). Collaborating with residency program leaders may allow us to use the program's existing competency-based evaluation system to better measure health equity-related behaviors.

## Conclusion

Although LHE is custom-designed for our local context, curricular features may be useful for others in the fields of medical education, health disparities, and leadership development. In particular, we propose several tactics that may further drive health equity educational efforts in the GME setting including integration of health equity content into leadership training, creation of structured opportunities for trainee introspection (3), and engaging pediatric trainees in content development. We are hopeful that this description of our approach and its early outcomes might provide insight to others heeding the call to action for enhanced health equity education in GME.

## Data Availability

The raw data supporting the conclusions of this article will be made available by the authors, without undue reservation.

## References

[1] Paul DW Knight KR Campbell A Aronson L. Beyond a moment - reckoning with our history and embracing antiracism in medicine. New Engl J Med. (2020) 383(15):1404–6. 10.1056/NEJMp2021812 32722907

[2] Braveman P Gruskin S. Defining equity in health. J Epidemiol Community Health. (2003) 57(4):254–8. 10.1136/jech.57.4.254 12646539 PMC1732430

[3] Siegel J Coleman DL James T. Integrating social determinants of health into graduate medical education: a call for action. Acad Med. (2018) 93(2):159–62. 10.1097/ACM.0000000000002054 29140918

[4] https://www.abp.org/sites/public/files/pdf/gen_peds_epa_14.pdf.

[5] Chary A Molina M Dadabhoy F Manchanda E. Addressing racism in medicine through a resident-led health equity retreat. Western J Emerg Med. (2021) 22(1):41–4. 10.5811/westjem.2020.10.48697 PMC7806337 33439802

[6] Denizard-Thompson N Palakshappa D Vallevand A Association of a health equity curriculum with medical students’ knowledge of social determinants of health and confidence in working with underserved populations. JAMA Network Open. (2021) 4(3):e210297. 10.1001/jamanetworkopen.2021.0297 33646312 PMC7921901

[7] Hernandez RG Hopkins A Dudas RA. The evolution of graduate medical education over the past decade: building a new pediatric residency program in an era of innovation. Med Teach. (2018) 40(6):615–21. 10.1080/0142159X.2018.1455969 29658367

[8] Sadowski B Cantrell S Barelski A O'Malley PG Hartzell JD. Leadership training in graduate medical education: a systematic review. J Grad Med Educ. (2018) 10(2):134–48. 10.4300/JGME-D-17-00194.1 29686751 PMC5901791

[9] Aysola J Myers JS. Integrating training in quality improvement and health equity in graduate medical education: two curricula for the price of one. Acad Med. (2018) 93(1):31–4. 10.1097/ACM.0000000000002021 29023244

[10] Wilkerson L Fung CC May W Elliott D. Assessing patient-centered care: one approach to health disparities education. J Gen Intern Med. (2010) 25(Suppl 2):S86–90. 10.1007/s11606-010-1273-5 20352499 PMC2847105

